# Gaucher disease with jawbone involvement: a case report

**DOI:** 10.1186/1752-1947-8-360

**Published:** 2014-11-05

**Authors:** Azadeh Ahmadieh, Fariborz Farnad, Parish P Sedghizadeh

**Affiliations:** 1 Ostrow School of Dentistry of USC, University of Southern California, Los Angeles, CA, USA; 2 USC Center for Biofilms, University of Southern California, 925 West 34th St. #4110, Los Angeles, CA 90089, USA

**Keywords:** Gaucher disease, Head and neck, Jawbone, Oral

## Abstract

**Introduction:**

Gaucher disease is an autosomal recessive systemic condition, and the most common of the lysosomal storage disorders. It is characterized by lipid accumulation in certain cells and organs, particularly macrophages, which appear on light microscopy as ’Gaucher cells’ or vacuolated lipid-laden reticuloendothelial cells. Long bone involvement is common in Gaucher disease, whereas craniofacial bone involvement is extremely rare. Reports confirming the diagnoses of Gaucher disease involving craniofacial bones by histopathologic evidence are even rarer.

**Case presentation:**

A 46-year-old Caucasian Ashkenazi Jewish woman with Gaucher disease presented with jawbone pain and lytic radiographic lesions of her mandible. Surgical biopsy of a mandibular lesion revealed Gaucher cells infiltrating the mandible, which correlated with radiographic and clinical findings, supporting a diagnosis of Gaucher disease with jawbone involvement.

**Conclusions:**

Lysosomal storage diseases can have head and neck manifestations, and bone involvement in Gaucher disease is common. Therefore, careful consideration of signs and symptoms and medical history, with a thorough review of systems, is important when evaluating patients with lysosomal storage disorders to rule out head and neck involvement of disease. Biopsy may be warranted in some cases for more definitive diagnosis of painful jawbone lesions and to rule out other odontogenic and non-odontogenic conditions in the differential diagnosis.

## Introduction

Gaucher disease is an autosomal recessive systemic lysosomal storage disorder that is panethnic but with higher prevalence in the Ashkenazi Jewish population. The disease is caused by a defect in the housekeeping gene lysosomal glucocerebrosidase on the first chromosome (1q22) [1]. Accumulation of lipid-laden macrophages in the reticuloendothelial system due to the deficiency of glucocerebrosidase enzyme causes the characteristic clinical features of Gaucher disease and results in the characteristic appearance of affected cells: an enlarged granular cytoplasm and round displaced nuclei [2].

Infiltration of Gaucher cells in tissues can be associated with systemic pathology such as hepatosplenomegaly, pancytopenia, skin pigmentation, neurologic symptoms, osteoporosis and severe bone pain [3]. Oral pathology can also be observed in Gaucher disease, and includes jaw lesions, delayed eruption of permanent teeth, oral yellow pigmentation, hyposalivation, dental pain and mobility, sinusitis, and osteomyelitis [4]. Bone involvement in Gaucher disease is seen in more than 90% of affected patients [4]. Long bone involvement is common, causing pain and restriction of mobility [5]. Craniofacial bones may also be affected, although more rarely reported. Jawbones have been described as being affected by Gaucher disease, and various radiographic features have been reported in the mandible (which is more commonly affected than the maxilla) such as soap-bubble or pseudocystic radiolucent lesions in the premolar-molar regions, loss of trabecularity, widening of bone marrow spaces, endosteal scalloping and loss of lamina dura around affected teeth [6]. Jawbones may also demonstrate generalized osteopenia and well-defined radiolucent lesions, with the resulting loss of cortication of the mandibular canal and sinus obliteration, bone expansion, and cortical perforation [4]. In some cases, apical root resorption is seen and might be related to accumulation of Gaucher cells in the apical region of teeth [7]. Importantly, jawbone involvement in Gaucher disease is usually asymptomatic [3,4].

Given the potential association with teeth, the clinical nonspecific presentation of pain, and the aforementioned radiographic features which could represent several odontogenic or non-odontogenic conditions, a diagnosis of Gaucher disease involving the jawbone can be diagnostically challenging and may warrant biopsy for definitive diagnosis. Only a few reports in the literature provide definite diagnosis of jawbone involvement by Gaucher disease as confirmed by biopsy rather than clinical and radiographic findings alone [4,8-14]. We report a unique case of Gaucher disease with jawbone involvement confirmed by biopsy.

## Case presentation

**Figure 1:**
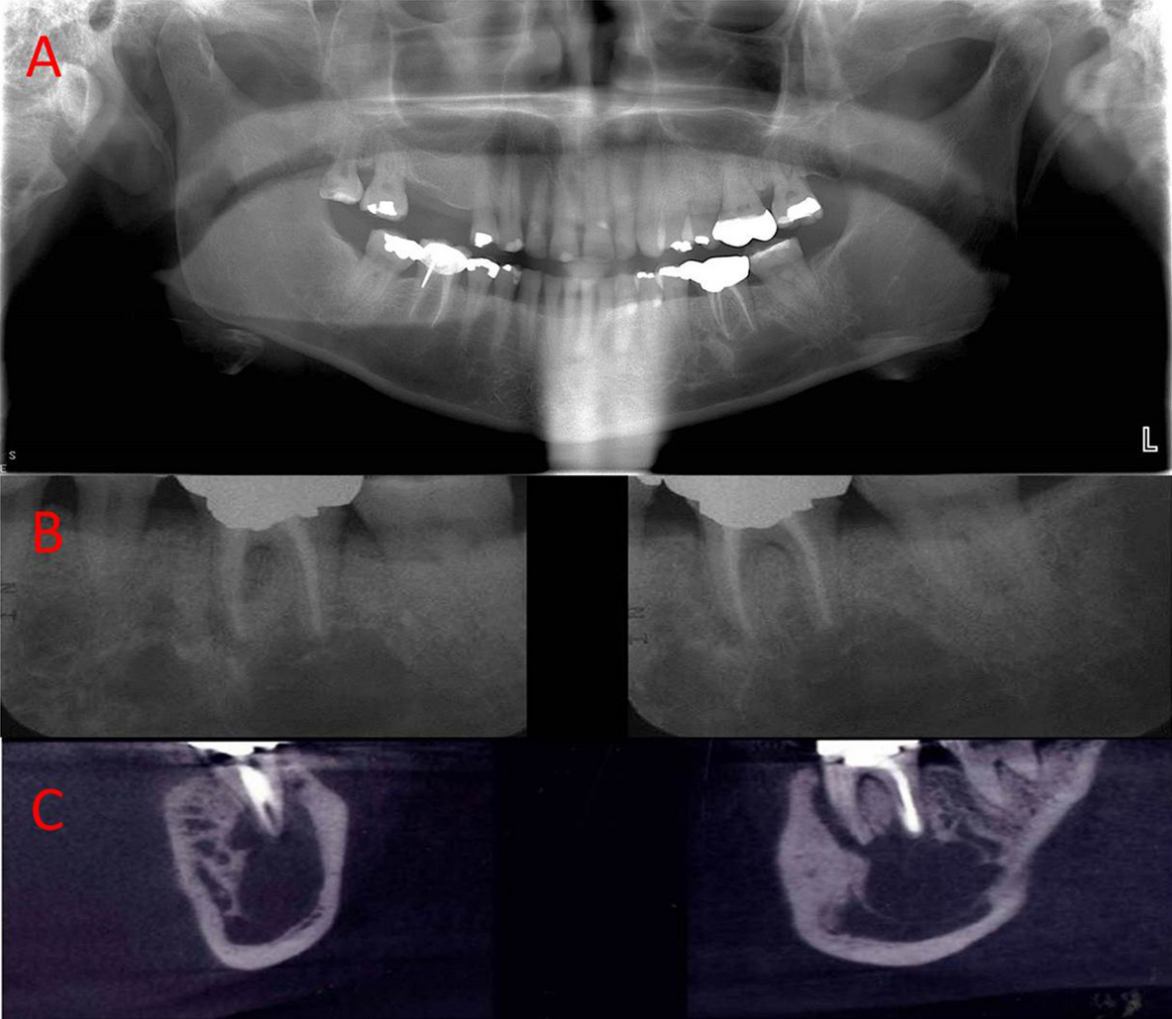
**Radiographic findings of the head and neck in a 46-year-old woman with Gaucher disease. A**. Panoramic radiograph demonstrating radiolucent or lytic lesions of the posterior mandibular, rarefaction of trabeculae, effacement of the mandibular canal and architecture of the antrum of the maxillary sinus with mild sinus opacification. **B**. Periapical X-rays from the region of the patient’s chief complaint demonstrating multiple relatively well-defined radiolucencies in the mandible with evidence of scalloping around teeth, corticated and curved peripheral margins in some areas, and minor root reabsorption. **C**. Cone-beam computed tomography scan of the mandible with coronal (left) and sagittal (right) reconstructions demonstrating the extent of the lytic lesion which cannot be appreciated with periapical films; enlarged marrow space, a multilocular appearance, and involvement of the periodontal ligament space with thinning and loss of lamina dura around the affected molars can be seen.

**Figure 2:**
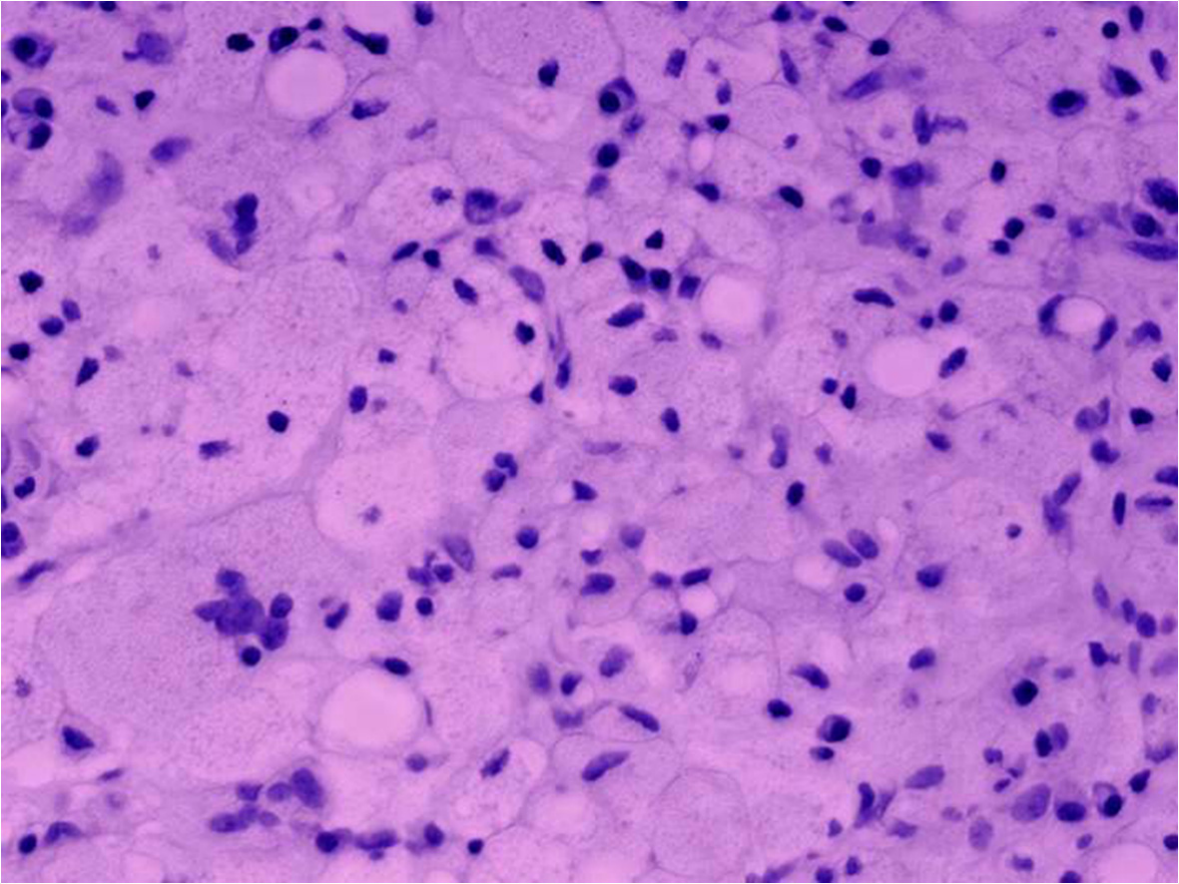
**Microscopic findings from surgical biopsy of the mandible revealed connective tissue infiltrated with numerous vacuolated lipid-laden reticuloendothelial cells or characteristic Gaucher cells with enlarged granular cytoplasm and round displaced nuclei.** Hematoxylin and eosin, 40× original magnification.

A 46-year-old Caucasian woman of Ashkenazi Jewish descent presented with the chief complaint of severe and constant throbbing pain in her left posterior mandible. She reported that her pain was aggravated by chewing, and started months before the initial consultation visit. Visual analog scale of pain was reported 7/10 at the day of the visit. Past medical history was remarkable for Gaucher disease, left hip replacement, gall bladder removal, herniorrhaphy, spleen abscess drainage, hepatitis C infection, and asthma. Social history was significant for 20 pack-years of cigarette smoking. Her family history was unremarkable for any hereditary or systemic diseases. Her medications included the oral antiresorptive drug alendronate for early osteoporosis associated with Gaucher disease (70mg weekly for 2 years), calcium supplements, loratadine, hydromorphone, esomeprazole, promethazine, levalbuterol, carisoprodol, fluticasone and ibuprofen. She reported that she had been on enzyme replacement therapy (ERT) intermittently for several years to treat her Gaucher disease which was diagnosed 20 years prior. She received intravenous injections twice a month for ERT and recounted fatigue after each infusion. She also reported receiving antibiotic and analgesic medications for her pain months ago but they were not helpful.Head and neck examination, cranial nerve examination and vital signs were within normal limits. Intraoral examination was completed using percussion testing, vitality testing and probing for evaluation of her mandibular posterior teeth in the region of the chief complaint. Her left mandibular first molar was endodontically treated 3 years prior and tested non-vital, while her remaining mandibular teeth were normal on vitality testing with evaluation of several opposing non-restored and non-carious teeth as internal controls. Gingival tissues in both jaws were non-inflamed and there was no gross evidence of plaque or calculus. Periodontal pockets in her mandible ranged from 4 to 6mm with no significant bony defects. Full mouth dental X-rays and a panoramic radiograph were taken and showed relatively well-defined radiolucent lesions in multiple regions of the mandible bilaterally, with a pseudocystic (multilocular) appearance; cone-beam computed tomography confirmed these findings and revealed a large lytic lesion of her left posterior mandible in the region of her chief complaint (Figure 1).Our clinical and radiographic differential diagnosis for her chief complaint included chronic apical periodontitis, radicular cyst, central giant cell granuloma, keratocystic odontogenic tumor, ameloblastoma, odontogenic fibroma, neuralgia/neuropathy or mandibular involvement of Gaucher disease given her medical history. Our findings were discussed with the patient and written consent was obtained for surgical biopsy of her left mandible with local anesthesia for more definitive diagnosis. A four-corner gingival flap buccally was reflected in her posterior left mandible extending from the canine to the second molar, and cortical bone was removed with a surgical bur to create a window for access to the lesion. Pathologic soft tissue was evident through the access window. The entire pathologic soft tissue along with the bone from the access window was submitted to the pathology laboratory for histological evaluation. Histopathological findings revealed infiltration of jawbone marrow with fibrous connective tissue containing abundant Gaucher cells (Figure 2). There was no evidence of abscess or neutrophils, granuloma or malignancy. Postoperative healing was uneventful, and the patient reported resolution of her chief complaint and symptomatology at 1-month follow-up. She was referred to her physician for consultation and further evaluation as related to her Gaucher disease status following our findings. She was again treated with ERT and at 1-year re-evaluation was still symptom free in her oral cavity.

## Discussion

Patients with Gaucher disease often complain of severe pain in various parts of their skeletal system, but rarely in the craniofacial or jawbones. Bender and Bender reported two cases of Gaucher disease with 13- and 60-years follow-up; in the first case mandibular lesions were present in the premolar-molar region and the involved teeth were vital [15]. These findings were consistent with our patient’s profile. Bender and Bender also reported that ERT improved the rarefaction of the mandible bilaterally with no evidence of osteolysis. However, some studies reported that ERT cannot reverse the necrotic and lytic changes in long bone and orthopedic intervention such as joint (hip, knee, shoulder) replacement is recommended [16]; our patient also had a history of hip joint replacement and long bone involvement by Gaucher disease. Bender and Bender stated that in the absence of clinical and laboratory examination, in the light of radiographic findings, no definitive diagnosis of jawbone involvement of Gaucher disease can be made without biopsy [15]; however, other studies have recommended biopsy only for cases where other conditions are suspected in the differential diagnosis [3], such as in the case presented here.

In the current case, radiographs of the head and neck showed lytic radiolucencies bilaterally in her mandible. Although the maxilla or sinuses can be involved in Gaucher disease, we could not appreciate such involvement in this case. A comprehensive review of the many radiographic jaw features in Gaucher disease was recently reported by Zeevi *et al*. [4]. Some of these findings (as detailed in Figure 1) were also present in our case. We also performed surgical biopsy of her mandible in the area of her chief complaint for more definitive diagnosis given the broad differential diagnosis. Surgical intervention resulted in the resolution of our patient’s chief complaint, which could be due to removal of pathologic tissue and healing at the site, or due to other factors not directly related to surgical intervention such as the natural course of her disease. As Gaucher cells were found in histological evaluation and similar radiolucencies were evident in the other sites of her mandible in radiographic evaluation, we can hypothesize that these lesions might be related to Gaucher disease also, but we cannot definitively make such a determination without biopsy from all involved sites. Since our patient was receiving oral bisphosphonate therapy, the potential existed for the development of jaw osteonecrosis following jawbone surgery. Although the risk of jaw osteonecrosis is minimal for oral bisphosphonate therapy as compared to intravenous therapy, particularly for a patient on only 2 years of therapy such as in this case, we nonetheless opted to minimize this risk by limiting the amount of bone surgery and biopsy.

The key to diagnosis of Gaucher lesions in the jawbones is thorough medical history and clinical examination, vitality and percussion testing of associated teeth when applicable to rule out odontogenic infections or lesions, radiographic evaluation, and in some cases histopathologic examination for definitive diagnosis.

## Conclusions

Lysosomal storage diseases can have head and neck involvement, and bone involvement in Gaucher disease is common. However, craniofacial bone involvement such as the jawbones is rarely reported. When involving the jawbones, Gaucher disease can mimic other odontogenic and non-odontogenic diseases and can be a diagnostic challenge. Therefore, familiarity with Gaucher disease and radiographic features in the jaws can facilitate accurate clinical diagnosis and management. Careful consideration of signs and symptoms and medical history, with a thorough review of systems, is important when evaluating patients with lysosomal storage disorders to rule out head and neck involvement of disease. Biopsy may be warranted in some cases for more definitive diagnosis of painful jawbone lesions since pain is a rare feature of Gaucher disease affecting the jawbones, and to rule out other odontogenic and non-odontogenic conditions in the differential diagnosis.

## Consent

Written informed consent was obtained from the patient for publication of this case report and any accompanying images. A copy of the written consent is available for review by the Editor-in-Chief of this journal.

## Competing interests

The authors declare that they have no competing interests.

## Authors’ contributions

All authors evaluated the patient, collected data, wrote and approved the final manuscript.
